# Evaluation of an alternative positive control strain of *Salmonella enterica* subsp*. enterica* serovar Typhimurium for microbial assays

**DOI:** 10.1371/journal.pone.0329363

**Published:** 2025-08-04

**Authors:** Yu-Si Lee, Su-Hyeon Joung, Yongchjun Park, Seung Hwan Kim, Soon Han Kim, Insun Joo, Eun Sook An

**Affiliations:** Division of Food Microbiology, National Institute of Food and Drug Safety Evaluation, Ministry of Food and Drug Safety, Cheongju, Republic of Korea; Islamic Azad University, IRAN, ISLAMIC REPUBLIC OF

## Abstract

Officially certified microbiological testing methods utilize positive control strains to enhance experimental reproducibility and ensure standardized procedures among experimenters. *Salmonella enterica* subsp. *enterica* serovar Typhimurium ATCC 14028 is designated as a positive control strain for microbiological testing by the International Organization for Standardization, the Korean Pharmacopoeia, and Ministry of Food and Drug Safety (MFDS) foodborne investigation methods. However, using such foreign strains involves complicated import procedures and significant financial burdens. In this study, we aimed to select a domestic isolate strain that can replace *S*. Typhimurium ATCC 14028. The target strains used were *S*. Typhimurium strains preserved in the Korean Culture Collection for foodborne pathogens (MFDS). To confirm the equivalent characteristics between the candidate strains and the positive control strain, biochemical and molecular characterization were performed according to the methods specified in ISO test methods, the Food Code, and the MFDS food poisoning investigation methods. After biochemical and molecular biological analyses on 19 *S*. Typhimurium strains, only those exhibiting equivalent characteristics underwent whole-genome sequencing. In the biochemical characterization, two strains showed different results in the citrate utilization test. Excluding these, the remaining 17 strains were subjected to molecular analysis (PCR), and all showed identical genetic profiles to the positive control strain. Ultimately, whole-genome sequencing of the 17 selected candidate strains revealed that strains 1004022 and 1004023 shared the same sequence type (ST19) as *S*. Typhimurium ATCC 14028, exhibited fewer than 20 SNPs, and showed 99.94% genomic homology. Therefore, *S*. Typhimurium MFDS 1004022 and 1004023 were proposed as suitable domestic alternative to the imported strain. It is anticipated that the distribution of these alternative strains to microbiological testing laboratories will contribute to food safety management by supporting microbial testing and analysis.

## Introduction

Officially certified test methods for detecting microorganisms in food include those of the International Organization for Standardization (ISO) and the Association of Official Analytical Chemists (AOAC). In Korea, test methods are specified in the Food Code, the Food Additives Code, and the Health Functional Food Code [1].

To increase the reproducibility of experiments and ensure the application of standardized methods among experimenters, officially certified microbiological test methods include positive controls [2–4]. Most of these positive controls are strains obtained from overseas resource banks such as the American Type Culture Collection (ATCC) and the National Collection of Type Cultures (NCTC). According to one report, 37,530 domestic microbial resources were secured in 2020, while a total of 2,016,211 resources (cumulative) were held. However, dependence on imported pathogen resources amounted to 88.7% [5]. In addition, Kim et al. (2020) reported that the supply of domestic resources was 751,490 (81.6%), 111,506 (12.1%), 44,547 (4.8%), and 13,320 (1.5%) for plants, human-derived materials, animals, and microorganisms, respectively [6]. In other words, while the distribution ratio of microorganisms among secured resources remains low, around 13,000 cases are still distributed annually. Considering the distribution rate of microbial resources and the high dependence on overseas resources, a significant amount of foreign currency is spent each year to purchase imported microbial strains. Furthermore, the enforcement of the Nagoya Protocol—which regulates access to and utilization of genetic resources essential for biotechnology research—has imposed practical challenges for researchers. By asserting national sovereignty over genetic resources, the Protocol imposes legal and financial restrictions on access and requires benefit-sharing for commercial applications [7]. As a result, researchers often face cumbersome import procedures, extended delivery times, and prohibitively high costs—sometimes up to 50 times higher than for domestic strains—when acquiring necessary foreign positive control strains [7,8]. Consequently, there is a growing need for research to identify domestic alternatives to imported positive control strains through comprehensive biochemical and genomic characterization.

*Salmonella enterica* subsp. *enterica* serovar Typhimurium ATCC 14028 is designated as a positive control strain in the ISO test methods, the microbial limit tests specified in the Korean Pharmacopoeia, and the confirmatory genetic test in food poisoning investigations by the Ministry of Food and Drug Safety (MFDS) [9–11]. This study aims to select a domestic isolate strain that can replace the strain *S.* Typhimurium ATCC 14028.

The Korean Culture Collection for Foodborne Pathogens of the MFDS, recognizing the importance of securing such resources, has been collecting and managing foodborne pathogens since 2012. These accumulated resources are actively utilized in various research projects, including the development of detection methods for foodborne pathogens and studies on their characteristics.

The characterization analysis of *Salmonella* Typhimurium in the Korean Culture Collection for Foodborne Pathogens of the MFDS was carried out, and a strain with high genomic homology among those with the same characteristics was selected as a replacement strain.

## Materials and methods

### Isolate collection

Nineteen *Salmonella enterica* subsp. *enterica* serovar Typhimurium candidate strains held in the Korean Culture Collection for foodborne pathogens (MFDS), isolated from domestic food and environments between 2014 and 2020 (Table 1). *Salmonella enterica* subsp. *enterica* serovar Typhimurium ATCC 14028 was purchased from American Type Culture Collection. The strains used in the experiment were stored in a freezer at −80°C, and subcultured on tryptic soy agar (TSA, Oxoid, London, England) at 37°C for 24 hours.

**Table 1:** List of the 19 candidate strains used in this study.

No.	Strain name	Year	Region	Source
1	1004022	2014	Jeonnam	Food environment (Dish towel)
2	1004023	2014	Jeonnam	Food environment (Cutting board)
3	1006894	2016	Daegu	Food (Pork entrails)
4	1006895	2016	Daegu	Food (Pork entrails)
5	1007324	2016	Kwangju	Food (Chicken)
6	1008085	2016	Daejeon	Food (Duck)
7	1011083	2018	Ulsan	Food (Duck)
8	1012040	2018	Gyeongbuk	Food (Duck)
9	1012372	2018	Gyeongnam	Food (Pork)
10	1013320	2019	Jeonbuk	Food (Duck)
11	1013362	2019	Busan	Food (Frozen tuna)
12	1013880	2019	Jeonnam	Food (Duck)
13	1016556	2020	Jeonnam	Food (Pork)
14	1016557	2020	Jeonnam	Food (Duck)
15	1016560	2020	Jeonnam	Food (Duck)
16	1016566	2020	Jeonnam	Food (Duck)
17	1017375	2020	Daejeon	Environment (Animal feces)
18	1017392	2020	Jeju	Food (Korean sausage)
19	1017394	2020	Jeonnam	Food (Duck)

### Biochemical and molecular characterization

Typical colonies were identified according to ISO and Food Code test methods. The media used for enrichment and isolation included Müller-Kauffmann tetrathionate-novobiocin broth (MKTTn broth), Rappaport-Vassiliadis medium with soya broth (RVS broth), and xylose lysine deoxycholate agar (XLD agar). Biochemical characterization was performed for triple sugar iron (TSI), urease, lysine decarboxylase, malonate, KCN, indole, methyl red (MR), voges proskauer (VP), and citrate in accordance with the test methods of the ISO, the Food Code, and the U.S. FDA’s Bacteriological Analytical Manual [9,12,13]. The TSI and KCN tests were performed using culture media (KisanBio Co., Ltd., Seoul, Korea). The citrate, urease, lysine decarboxylase, and malonate tests were performed using a VITEK 2 automated system (BioMérieux Inc., France) while the indole, MR, and VP tests were performed using a commercial kit (KisanBio Co., Ltd., Seoul, Korea). All of the tests were subject to the protocols provided by the manufacturers. Molecular biological characterization was performed according to the method of investing the causes of foodborne by the Ministry Food and Drug Safety, targeting *S.* Typhimurium (*typh*), *Salmonella* serogroup C2 (*had*), tetrathionate respiration (*ttr*), and invasion protein (*invA*) genes to determine whether they were detected [11]. Genomic DNA was extracted using the MagListo^TM^ 5M Genomic DNA Extraction Kit (Bioneer, Daejeon, Korea) in accordance with the manufacturer’s protocol, and then PCR and whole genome sequencing were performed. A conventional PCR reaction mixture was prepared by mixing 5 μL of DNA and 1 μL each of the forward and reverse primers (10 pmol/μL) with AccuPower PCR Premix (Bioneer) to make a final volume of 20 μL. The PCR reaction conditions and primer sequencing information are shown in Table 2.

**Table 2:** Primer sequence and PCR conditions used for *Salmonella* spp.

Target gene	Sequence (5’ → 3’)	Product size (bp)	Amplification conditions
*typh*	(F)TTG TTC ACT TTT TAC CCC TGA A (R)CCC TGA CAG CCG TTA GAT ATT	401	Initial denaturation: 95°C (2 min) 30 cycles of 95°C (1 min), 57°C (1 min), 72°C (2 min) Final extension: 72°C (5 min)
*had*	(F)ACC GAG CCA ACG ATT ATC AA (R)AAT AGG CCG AAA CAA CAT CG	502	

Gene amplification was performed for each gene using a C1000 Touch^TM^ Thermal Cycler (Bio-Rad Laboratories, Inc., Singapore), and the results were confirmed by electrophoresis on a 1.5% agarose gel. The primer/probe sequences and experimental conditions of the real-time PCR are shown in Table 3. Amplification was conducted using ABI 7500 Fast Real-time PCR System (Applied Biosystems, Waltham, MA, USA).

**Table 3:** Primer/probe sequence and real-time PCR conditions used for *Salmonella* spp.

Target gene	Sequence (5’ → 3’)	Amplification conditions
*invA*	(F)GAA TCC TCA GTT TTT CAA CGT TTC (R)CGA ATT GCC CGA ACG TGG CGA (P)FAM-CTC TTT CGT CTG GCA TTA TCG ATC AGT ACC AG-BHQ1	50°C (2 min), 1 cycle of 95°C (10 min), 40 cycles of 95°C (15 sec), 60°C (1 min)
*ttr*	(F)CTC ACC AGG AGA TTA CAA CAT GG (R)AGC TCA GAC CAA AAG TGA CCA TC (P)Cy3-CAC CGA CGG CGA GAC CGA CTT T-BHQ3	

## Comparative genomics

### Whole genome sequencing and genome assembly

The concentration of extracted gDNA was measured using a Qubit^TM^ 3.0 Fluorometer (Life Technologies, Carlsbad, CA, USA) to set the final concentration to 30 ng. Sequencing libraries were constructed using Nextera^TM^ DNA Flex and a Nextera DNA Flex Library Prep Kit (Illumina, San Diego, CA, USA). The amplified libraries were identified using a Bioanalyzer 2100 instrument (Agilent Technologies, Waldbronn, Germany) and a QubitTM 3.0 Fluorometer (Life Technologies, Carlsbad, CA, USA). Sequencing was performed using a MiSeq sequencing system (Illumina) and MiSeq Reagent Kit v3 (600 cycles) (Illumina) [14]. Raw reads (FASTQ sequence files) were subjected to assembly using SPAdes v4.0.0 [15] (S1 Table). Sequence data have been submitted to the publicly accessible NCBI (https://ncbi.nlm.nih.gov, accessed on 12 February 2025).

### Phylogenetic analyses

Analyses of whole-genome multilocus sequence typing (wgMLST), single nucleotide polymorphism (SNP), and orthologous average nucleotide identity (OrthoANI) were performed to compare the genetic homology between the positive control strain and the candidate strains. Whole-genome multilocus sequence typing (wgMLST) was performed with a BioNumerics v7.5 (Applied Maths, Sint-Martens-Latem, Belgium) calculation engine using the default settings. The reads were de novo assembled by wgMLST analysis of assembly-free, and assembly-based allele calls were performed. Categorical coefficients were used to define similarity levels and the unweighted pair group method with arithmetic mean (UPGMA) was used as the clustering algorithm [16,17]. Single nucleotide polymorphism (SNP) was performed using the National Genome Information Network for Foodborne Pathogen (NGIN-F) of the Ministry of Food and Drug Safety (https://nginf.nifds.go.kr/cm/main.do). The orthologous average nucleotide identity (OrthoANI) was analyzed using the Orthologous Average Nucleotide Identity Tool (http://www.ezbiocloud.net/sw/oat) [15,18].

## Results

### Biochemical and molecular characterization

Typical *Salmonella* colonies on XLD agar, characterized by red colonies with black centers, were observed in all 19 strains. In the biochemical characterization, two strains showed different results in the citrate test. In the genetic analysis, 17 strains (excluding the two with different citrate results) showed the same results as *S*. Typhimurium ATCC 14028, and were selected as final candidate strains (Table 4).

**Table 4:** Detailed biochemical and molecular characteristics of *Salmonella* Typhimurium ATCC 14028 and candidate strains. Two strains different results in the citrate test. All other characteristics were identical to those of the positive control strain.

No.	Strain name	Biochemical analysis (n = 19)									Genetic analysis (n = 17)			
TSI	Urease	Lysine	Malonate	KCN	Indole	MR	VP	Citrate	*typh*	*ttr*	*had*	*invA*
1	ATCC 14028	+	–	+	–	–	–	+	–	+	+	+	–	+
2	1004022	+	–	+	–	–	–	+	–	+	+	+	–	+
3	1004023	+	–	+	–	–	–	+	–	+	+	+	–	+
4	1006894	+	–	+	–	–	–	+	–	+	+	+	–	+
5	1006895	+	–	+	–	–	–	+	–	+	+	+	–	+
6	1007324	+	–	+	–	–	–	+	–	–	-^a^	-^a^	-^a^	-^a^
7	1008085	+	–	+	–	–	–	+	–	+	+	+	–	+
8	1011083	+	–	+	–	–	–	+	–	+	+	+	–	+
9	1012040	+	–	+	–	–	–	+	–	+	+	+	–	+
10	1012372	+	–	+	–	–	–	+	–	+	+	+	–	+
11	1013320	+	–	+	–	–	–	+	–	+	+	+	–	+
12	1013362	+	–	+	–	–	–	+	–	+	+	+	–	+
13	1013880	+	–	+	–	–	–	+	–	+	+	+	–	+
14	1016556	+	–	+	–	–	–	+	–	–	-^a^	-^a^	-^a^	-^a^
15	1016557	+	–	+	–	–	–	+	–	+	+	+	–	+
16	1016560	+	–	+	–	–	–	+	–	+	+	+	–	+
17	1016566	+	–	+	–	–	–	+	–	+	+	+	–	+
18	1017375	+	–	+	–	–	–	+	–	+	+	+	–	+
19	1017392	+	–	+	–	–	–	+	–	+	+	+	–	+
20	1017394	+	–	+	–	–	–	+	–	+	+	+	–	+

TSI: Triple sugar iron, MR: Methyl red, VP: Voges proskauer, − : Negative, + : Positive, -^a^: The strain excluded from molecular biological tests

### Comparative genomics

**Fig 1:**
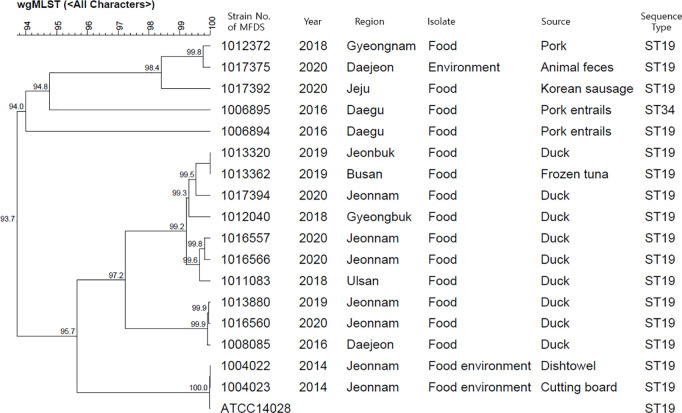
wgMLST dendrogram showing the phylogenetic relationships between *S*. Typhimurium ATCC 14028 and 17 candidate strains. Branch lengths represent genetic distance based on allelic differences, with higher values indicating greater divergence.

**Fig 2:**
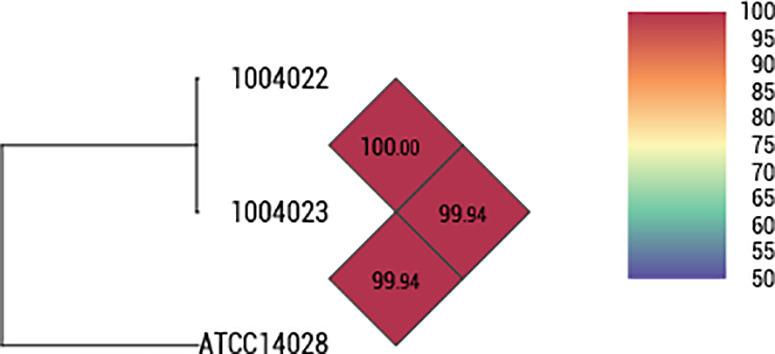
Heatmap generated based on orthologous average nucleotide identity (OrthoANI) values calculated between *S.* **Typhimurium ATCC 14028 and candidate strains.** OrthoANI values indicate genetic similarity between genomes (red indicates high similarity, whereas purple indicates lower similarity).

The genetic homology analysis of the 17 strains with biochemical characteristics equivalent to those of *S*. Typhimurium ATCC 14028 was performed using wgMLST and SNP. The wgMLST analysis revealed a homology range of 93.7% to 100% among all strains. Regarding sequence type (ST), the positive control strain was identified as ST19. Similarly, 16 out of 17 candidate strains were classified as ST19, while one strain belonged to ST34 (Fig 1). SNP analysis showed that among the candidate strains, MFDS 1004022 and 1004023 exhibited the highest similarity to the positive control strain, with fewer than 20 SNP differences (S2 Table). The sequence similarity between the positive control strain (*S*. Typhimurium ATCC 14028) and the alternative candidate strains (MFDS 1004022 and 1004023) were analyzed using the OrthoANI program. The OrthoANI values indicated a high genomic homology of 99.94% (Fig 2). Overall, the gene homology analysis confirmed a high genomic similarity between the positive control strain (*S*. Typhimurium ATCC 14028) and the domestically isolated strains MFDS 1004022 and 1004023. Therefore, these strains are proposed as suitable domestic replacements for the imported *S*. Typhimurium ATCC 14028 strain.

## Discussion

Practitioners from Korean testing and inspection agencies purchase and utilize positive control strains to ensure the reliability of experimental results. Domestically and internationally certified microbiological test methods primarily specify strains from overseas resource banks, such as the ATCC and NCTC, as positive control strains. However, strains specified in official test methods are costly and their import procedures are complex, creating a significant burden for domestic researchers. This study aimed to identify domestic isolates with characteristics equivalent to those of positive control strains specified in domestic and international test methods for use in laboratories, method development, educational materials, and research. The *S.* Typhimurium ATCC 14028 strain is used as a positive control strain in ISO method for microbial analysis and is also referenced in the Korean Pharmacopoeia. Previous studies have shown that *S.* Typhimurium ATCC 14028 is commonly utilized in biochemical reaction tests and genetic analyses using PCR. To replace the ATCC strain, comparative experiments were conducted with MFDS strains isolated in Korea. The results revealed that the morphological, biochemical, and molecular characteristics of MFDS strains 1004022 and 1004023 were identical to those of the positive control strain. Furthermore, whole-genome sequence comparison revealed a high level of genomic similarity, with an OrthoANI value of 99.94%, shared sequence type (ST19), and fewer than 20 SNPs. For OrthoANI values, a similarity of 94% or higher indicates that the genome composition is nearly identical within the same species, as it measures DNA sequence similarity between two strains at the species level [19,20]. Pightling et al. [21] indicate that an SNP distance of less than 21 strongly supports the genetic identity or very close relatedness of two isolates, suggesting a common source of origin. The *S.* Typhimurium MFDS 1004022 and 1004023 strains, isolated from environmental sources such as dish towels and cutting boards in 2014, are proposed as potential replacement strains for *S.* Typhimurium ATCC 14028.

An et al. [8] conducted a study to identify a domestic strain capable of replacing *Staphylococcus aureus* ATCC 6538P, as referenced in the Korean Pharmacopoeia. From the National Culture Collection of Pathogens (NCCP), a strain (NCCP 16830) was identified with characteristics equivalent to those of the ATCC 6538P reference strain, and its potential as a replacement strain was proposed.

The United States Pharmacopeia (USP) specifies that replacement strains proven to be identical to reference strains can be used as substitutes [8,22]. In this study, the proposed alternative strains demonstrated both identity and specificity to the positive control strain through phenotypic, biochemical, genetic, and whole-genome analyses. In particular, the high genetic similarity revealed by whole-genome analyses, including OrthoANI and SNP comparisons, indicates their equivalence to the positive control strain. Although this study did not include the process of verifying whether the proposed alternative strains can fully substitute ATCC 14028 in actual microbiological test assays, these strains were previously isolated and identified from food and environmental samples during foodborne outbreak investigations. Given that they have already undergone biochemical and genetic identification, their reproducibility was considered demonstrated. Nevertheless, additional validation—such as viability and stability tests under various environmental conditions—will be needed before distribution.

The strains selected in this study have been validated for use as alternative positive control strains in microbiological testing under ISO standards and the Korean Food Code. However, to apply them as alternatives in other sectors, further functional validation will be required in accordance with the relevant test methods, including the general testing methods outlined in the Korean Pharmacopoeia and the sterilization and disinfection test methods in the Food Additives Code.

*S*. Typhimurium MFDS 1004022 and 1004023 are preserved in The Korean Culture Collection for Foodborne Pathogens of the Ministry of Food and Drug Safety (MFDS), which is designated as a specialized pathogen resource bank under the “Act on the Management and Utilization of Pathogen Resources” [23]. These alternative strains are available for distribution to testing and inspection agencies for use in microbiological testing and analysis, which will contribute to food safety management.

This is expected to increase the utilization of domestic strains, significantly reduce the costs and time associated with importing foreign strains, and decrease dependence on overseas resources, thereby alleviating the burden on researchers. Moreover, the use of domestic microbial resources may enhance the efficiency of microbiological testing and contribute to strengthening the national bioresource infrastructure.

## Nucleotide sequence accession numbers

The draft genome sequences of *Salmonella* Typhimurium MFDS 1004022 and *Salmonella* Typhimurium MFDS 1004023 have been deposited with GenBank under the accession numbers JBLQAJ000000000 and JBLQAK000000000, respectively.

## Supporting information

S1 Table Statistics of the assembly results of the whole genome of 17 *S.* Typhimurium strains.
(DOCX)

S2 Table The number of SNP differences in 17 *S*. Typhimurium strains. (XLSX)

## References

[1] Bang HJ, Lee SH, Kim YS, Kim AP, Lee WJ, Ha SD. International trends of official analytical methods for food microorganisms and the principles of detection methods. Safe Food. 2015;10(3):42–51.

[2] Nysten J, Sofras D, Van Dijck P. One species, many faces: The underappreciated importance of strain diversity. PLoS Pathog. 2024;20(1):e1011931. doi: 10.1371/journal.ppat.1011931 38271302 PMC10810500

[3] Petersen EJ, Nguyen A, Brown J, Elliott JT, Clippinger A, Gordon J, et al. Characteristics to consider when selecting a positive control material for an in vitro assay. ALTEX. 2021;38(2):365–76. doi: 10.14573/altex.2102111 33637998

[4] Hornung BVH, Zwittink RD, Kuijper EJ. Issues and current standards of controls in microbiome research. FEMS Microbiol Ecol. 2019;95(5):fiz045. doi: 10.1093/femsec/fiz045 30997495 PMC6469980

[5] MSIT (Ministry of Science and ICT). 2021 National life science research resource management and utilization implementation plan. 2020. pp. 20–52.

[6] Kim C-M, Park K-M, Cho K-H, Jo G-H, Jin T-E. The Current Status and Policy Measure of Biological Research Resources in Korea. jepa. 2020;28(4):91–112. doi: 10.15301/jepa.2020.28.4.91

[7] Lee J, An M, Chang Y-H. Understanding the Access and Benefit-Sharing of Genetic Resources for Microbiology Researchers. Kor J Microbiol Biotechnol. 2021. doi: 10.48022/mbl.2104.04015

[8] An YW, Choi YS, Yun M-R, Choi C, Kim SY. Characterization and validation of an alternative reference bacterium Korean Pharmacopoeia Staphylococcus aureus strain. J Microbiol. 2022;60(2):187–91. doi: 10.1007/s12275-022-1335-5 34994956 PMC8739380

[9] ISO (International Organization for Standardization). Horizontal method for the detection, enumeration and serotyping of *Salmonella* - Part 1: Horizontal method for the detection of Salmonella spp. ISO/TR 6579-1:2017; 2022.

[10] MFDS (Ministry of Food and Drug Safety). The Korean Pharmacopoeia, 12th edn. 2020.

[11] MFDS (Ministry of Food and Drug Safety). Manual for the Detection of Foodborne Pathogens in Outbreaks. 2020.

[12] Ministry of Food and Drug Safety. Korean Food Code. MFDS, Republic of Korea. 2020.

[13] Andrews WH, Wang H, Jacobson A, Ge B, Zhang G, Hammack T. BAM Chapter 15: Salmonella. FDA. 2020.

[14] Lee W, Kim M-H, Sung S, Kim E, An ES, Kim SH, et al. Genome-Based Characterization of Hybrid Shiga Toxin-Producing and Enterotoxigenic Escherichia coli (STEC/ETEC) Strains Isolated in South Korea, 2016-2020. Microorganisms. 2023;11(5):1285. doi: 10.3390/microorganisms11051285 37317259 PMC10223106

[15] Oh JS, Roh DH. Draft genome sequences of Sabulilitoribacter multivorans KCTC 32326T and Sabulilitoribacter arenilitoris KCTC 52401T. Korean J Microbiology. 2022;58(3):205–7. doi: 10.7845/kjm.2022.2054

[16] Rokney A, Valinsky L, Moran-Gilad J, Vranckx K, Agmon V, Weinberger M. Genomic Epidemiology of Campylobacter jejuni Transmission in Israel. Front Microbiol. 2018;9:2432. doi: 10.3389/fmicb.2018.02432 30386311 PMC6198274

[17] Papić B, Diricks M, Kušar D. Analysis of the Global Population Structure of Paenibacillus larvae and Outbreak Investigation of American Foulbrood Using a Stable wgMLST Scheme. Front Vet Sci. 2021;8:582677. doi: 10.3389/fvets.2021.582677 33718463 PMC7952629

[18] Lee I, Ouk Kim Y, Park S-C, Chun J. OrthoANI: An improved algorithm and software for calculating average nucleotide identity. Int J Syst Evol Microbiol. 2016;66(2):1100–3. doi: 10.1099/ijsem.0.000760 26585518

[19] Crosby KC, Rojas M, Sharma P, Johnson MA, Mazloom R, Kvitko BH, et al. Genomic delineation and description of species and within-species lineages in the genus Pantoea. Front Microbiol. 2023;14:1254999. doi: 10.3389/fmicb.2023.1254999 38029109 PMC10665919

[20] Konstantinidis KT, Tiedje JM. Genomic insights that advance the species definition for prokaryotes. Proc Natl Acad Sci U S A. 2005;102(7):2567–72. doi: 10.1073/pnas.0409727102 15701695 PMC549018

[21] Pightling AW, Pettengill JB, Luo Y, Baugher JD, Rand H, Strain E. Interpreting Whole-Genome Sequence Analyses of Foodborne Bacteria for Regulatory Applications and Outbreak Investigations. Front Microbiol. 2018;9:1482. doi: 10.3389/fmicb.2018.01482 30042741 PMC6048267

[22] The Unites States Pharmacopoeia. Antibiotics-Microbial Assays, USP43-NF38-6488, pp. 44.

[23] Korea Disease Control and Prevention Agency. Act on the management and utilization of pathogen resources Law No. 18614, Republic of Korea. 2022.

