# Supplementary material for: Evaluation of an alternative positive control strain of Salmonella enterica subsp. enterica serovar Typhimurium for microbial assays
Source: PLoS One. 2025 Aug 4;20(8):e0329363. doi: 10.1371/journal.pone.0329363 (PMC12321117; doi:10.1371/journal.pone.0329363)
Supplement: S1 Table — Typhimurium strains. (DOCX) [file pone.0329363.s001.DOCX]

**S1 Table. Statistics of the assembly results of the whole genome of 17 *S*. Typhimurium**

**strains.**

| No. | Strain Name | Genome size (bp) | Coverage | Contig Total Num (>500 bp) | GC% |
| --- | --- | --- | --- | --- | --- |
| 1 | 1004022 | 4,917,090 | 170.1 | 48 | 52.2 |
| 2 | 1004023 | 4,906,507 | 168.7 | 52 | 52.2 |
| 3 | 1006894 | 5,098,930 | 129.4 | 53 | 52.0 |
| 4 | 1006895 | 4,978,564 | 163.4 | 45 | 52.1 |
| 5 | 1008085 | 4,937,034 | 182.1 | 49 | 52.1 |
| 6 | 1011083 | 4,943,766 | 142.7 | 64 | 52.1 |
| 7 | 1012040 | 4,945,178 | 122.5 | 60 | 52.1 |
| 8 | 1012372 | 4,930,722 | 107.2 | 55 | 52.2 |
| 9 | 1013320 | 4,902,206 | 100.5 | 60 | 52.1 |
| 10 | 1013362 | 4,921,732 | 128.8 | 73 | 52.1 |
| 11 | 1013880 | 4,928,405 | 116.4 | 55 | 52.1 |
| 12 | 1016557 | 4,939,382 | 104.0 | 53 | 52.1 |
| 13 | 1016560 | 4,911,595 | 82.1 | 50 | 52.2 |
| 14 | 1016566 | 4,938,752 | 89.4 | 52 | 52.1 |
| 15 | 1017375 | 4,930,977 | 93.1 | 59 | 52.2 |
| 16 | 1017392 | 5,014,830 | 88.6 | 71 | 52.1 |
| 17 | 1017394 | 4,946,714 | 137.4 | 63 | 52.1 |
